# LGBTQIA+STEM Day 2022: an interview with Kenro Kusumi and Sarah Gordon

**DOI:** 10.1038/s42003-022-04201-2

**Published:** 2022-11-18

## Abstract

To commemorate LGBTQIA+ STEM Day this year, we reached out to faculty in institutional and national leadership roles to discuss their academic journeys, the importance of having queer role models and ways to better support LGQTBIA+ researchers.

Dr. Kenro Kusumi (he/him) is the Dean of Natural Sciences and Professor in the School of Life Sciences at Arizona State University.

**Figure:**
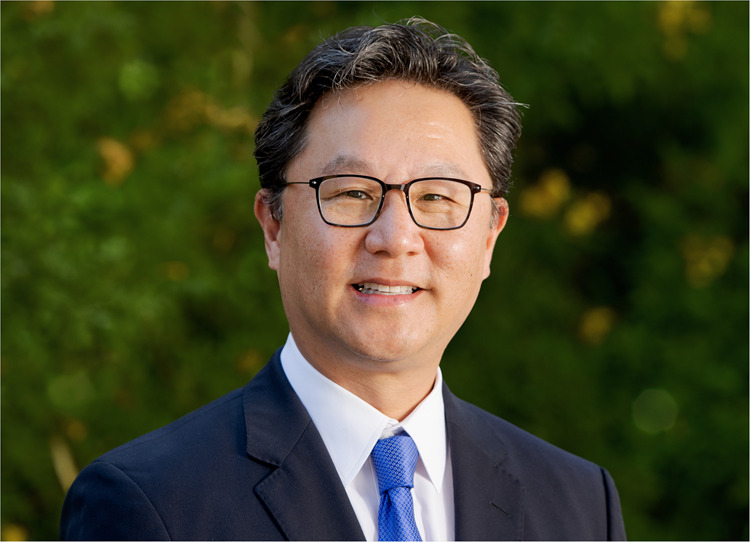
Kenro Kusumi

Dr. Sarah Gordon (she/her) is the Head of the Presynaptic Physiology Laboratory at the Florey Institute of Neuroscience and Mental Health and co-chair of the Victorian chapter of QueersInScience in Australia.

**Figure:**
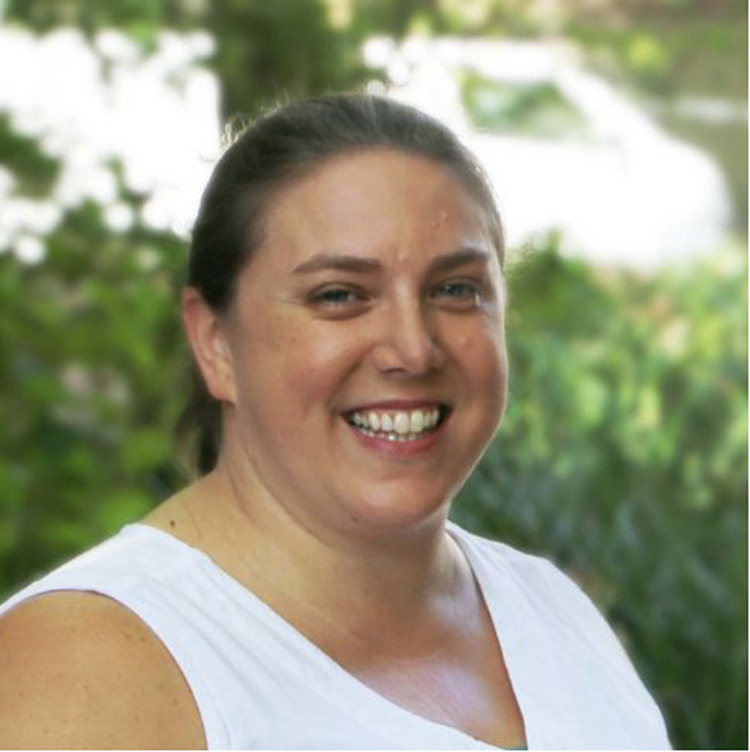
Sarah Gordon

Please tell us a little bit about your academic background, as well as your current research?

**Sarah Gordon (SG):** After completing my undergraduate degree in Biomedical Science and my Ph.D. in Medical Biochemistry at the University of Newcastle in Australia, I ended up on the other side of the world at the University of Edinburgh to undertake a postdoc in synaptic neurobiology. I was fortunate there to be involved in a lot of interesting projects, meet some really fantastic collaborators and start the work that now forms a major foundation of my lab at the Florey Institute of Neuroscience and Mental Health in Melbourne (Australia). My team is really interested in the molecular machinery that controls neurotransmission, and what happens when that machinery becomes dysfunctional.

**Kenro Kusumi (KK):** I am a genome biologist who has worked on projects ranging from human disease genetics to conservation genomics. I was raised in North Carolina and went to Harvard College, where I met my husband, who studies the complex behavior of social insects. My college and graduate work took place as the HIV/AIDS epidemic was unfolding, and, as a gay scientist, I was committed to advancing AIDS education and doing what I could to speed up HIV research. In fact, my first publications were on HIV virology and vaccines, and this experience turned out to be useful during the COVID-19 pandemic. My husband and I navigated going to the UK for our postdoctoral fellowships, and I returned to start a faculty position at the University of Pennsylvania and Children’s Hospital of Philadelphia, working to identify genes responsible for congenital forms of scoliosis. We were finally able to both find academic positions at Arizona State University in 2006, and we’ve been in Phoenix since then. Currently, my group is using the power of genomics to help in the conservation of reptiles, particularly turtles. Like many reptiles, half of the world’s turtle species are threatened with extinction, and my lab has been working closely with state and federal agencies to advance their conservation. We are working on population studies building on genome sequencing, including the threatened Mojave desert tortoise (*Gopherus agassizii*), the Sonoran desert tortoise (*G. morafkai*), and the Texas tortoise (*G. berlandieri*). I feel that this work has an impact in helping to ensure that these species survive into the 22^nd^ century.

Who has been a role model or key mentor in STEM (LGBTQIA+ or otherwise) that has had an impact on your career?

**SG:** I’ve been really lucky to have some really fantastic mentors throughout my career. My Ph.D. supervisor Phil Dickson really allowed me to grow as an independent scientist, and during my postdoc my lab head Michael Cousin just threw every opportunity at me he could. They were both really different leaders, and I’ve drawn a lot of inspiration from them when creating my own research group. But if I look back, the person who probably inspired me the most was my grade 6 teacher Michael Falkiner – he inspired a real love of science and passion for learning in me that is still at my core today. My favourite thing about science is that you are always learning (it is literally your job) and there’s that fantastic moment where you are the first person in the whole history of humanity to know a thing. I think he kindled that thirst for knowledge and discovery in me.

**KK:** When I was in college and graduate school, there really were not any out LGBTQIA+ role models, given the hostile environment of the 1980s. With the advances for LGBTQIA+ rights in the past decades, I feel strongly that it is important for students to see members of our community in all aspects of STEM training. I learned so much about balancing a career in research with administration through my graduate and postdoctoral advisors, who balanced being administrators as well as leading scientists. Also from informal mentors, including a respected geneticist who went on to become a university president; I appreciated being able to ask her about that process.

What does it mean to you, to be queer in STEM?

**SG:** Gosh, I wish it meant nothing to me, but that’s unfortunately not the case. As a junior researcher, I don’t think I knowingly came across any people in leadership positions in STEM who were queer. There was one other queer lab head when I started my own lab, and when he left, I suddenly realised that I was it – and I felt this huge weight of responsibility and isolation. Luckily at about the same time I started networking with other queer scientists from other institutions, which helped me navigate some of that wave of anxiety and pressure. I now have quite a few queer people in my team (about half of my lab is queer) and I get a sense that we’re changing things for the better for the next generation. If anything, being queer has probably made me step up into leadership roles a little more actively, because of that sense of responsibility.

**KK:** Like the opening of Dickens’s *Tale of Two Cities*, this is both an exciting time to be queer in STEM but also one of anxious uncertainty. For the first time in 2022, we have two Nobel Prize awardees who are members of the LGBTQIA+ community (Svante Pääbo and Carolyn Bertozzi). However, at the same time, there is legislation in many states that are targeting information and support services available to LGBTQIA+ students. I hope that the increasing visibility of LGBTQIA+ scientists will demonstrate our contributions to STEM education and research. As scientists who are different from the majority, can we ask questions and investigate areas that others have not explored before?

What do you see as key barrier(s) to LGBTQIA+ researchers in STEM fields?

**SG:** This is so hard to say, mostly because we lack good data, particularly in Australia. So I think that’s our first issue, is that we don’t even know what the barriers are. But from my personal experience, lack of visible role models is a big one. If I give a lecture or a talk about being LGBTQIA+ in STEM, then I almost always get a queer student or postdoc come up to me afterwards to tell me how powerful it is to see queer people in leadership roles. Of course, if you are trans or non-binary in STEM then there systematic issues at play too, which just increases the barrier to entry even more.

**KK:** It is so important to develop the social and informational networks to help LGBTQIA+ researchers navigate their careers, and we still have far to go in establishing these communities. Unfortunately, there are many places where the work environments can contribute to stress and not allow people to be comfortably out. Researchers often move frequently during their education and jobs and finding health care and other basic services responsive to our needs can be challenging, especially outside of major urban areas. Science can involve the need to move or travel internationally to advance our careers, and the different levels of same-sex relationship recognition can make this challenging.

Kenro: As a Dean at Arizona State University (ASU), you are in a unique position to influence university policy. Are there any initiatives you have taken to promote LGBTQIA+ issues or an overall more inclusive academic environment?

**KK:** Inclusion not only benefits the scientific community but also the scientific process. Science is how we discover the world around us, and this process is tremendously enriched by having scientists who don’t think in the same ways as the majority of people. Our different experiences, backgrounds, and perspectives, including as LGBTQIA+ researchers, are critical as we adapt to a rapidly changing planet in the 21^st^ century. Towards building greater inclusion, I have been involved in mentorship at many levels, ranging from serving as faculty advisor to our LGBTQIA+ graduate student group to being a mentor to students through university and the oSTEM national network. As an individual professor, I am out in class and make sure my students can indicate their preferred name and pronouns and give clear instructions to adjust their preferred name in the learning platform. As dean, I sponsor seed grants to support our creative faculty and staff to develop new projects to advance diversity, equity, and inclusion. I have taken every opportunity to support our amazing faculty who are carrying out research on inclusive STEM education, including the academic success of LGBTQIA+ students. We have established a scholarship to advance graduate student leadership in LGBT-related research. I’m grateful for ASU’s charter, which focuses on inclusion: we are “measured not by whom we exclude, but rather by whom we include and how they succeed.” My efforts to create an environment of belonging for LGBTQIA+ students, faculty and staff in the natural sciences division is bolstered by this charter. There’s so much more we could do, and I am always open to ideas and projects that will build a more inclusive environment.

Sarah: In addition to your role at the Florey Institute, you are also a co-chair for the Victorian chapter of QueersInScience. How would you describe this organization someone who has not heard of it before?

**SG:** We’re a grassroots organisation that is made up of queer people from across the STEM sectors, which aims to build community and improve support for LGBTQIA+ people in STEM in Australia. The organisation is built on a foundation of 5 pillars – visibility, advocacy, networking, education and intersectionality. Basically we’re a bunch of queer nerds trying to support other queer nerds. At our heart, we’re trying to make a difference – and the journey a little easier – for queer people in STEM.

Sarah: What are some of the initiatives you have overseen for QueersInScience? What are ways for interested researchers to get involved?

**SG:** I’m really proud of what we’ve achieved in QueersInScience. We’ve run a range of professional development and networking events for LGBTQIA+ people in STEM, as well as social events with the idea that this helps build bridges between queer researchers and allows people to find mentors who can really understand their life experience and help provide guidance and support. I think the event that I’m most proud of is holding the first symposium for LGBTQIA+ people in STEM for LGBTQIA+ STEM Day. We had so much positive feedback about how important the day was for people – that they really felt like they had found a sense of community for the first time. We’re always looking for more people to be part of the committee, and we have one based in each state in Australia. The best way to get in contact with us is via our website (https://queersinscience.org.au/). If you’re looking for your “people” then you might find them here.

More broadly, what do you both think academic institutions should be doing to help LGBTQIA+ researchers or trainees?

**KK:** It is important for administrators to meet with LGBTQIA+ students, staff and faculty and listen. What would allow them to be more successful at their institution? Administrators should advocate for the needs of our LGBTQIA+ students, especially given the challenges that they are facing. Administrators should take the opportunities to learn more about LGBTQIA+ issues; there are resources available from a number of societies, organizations, and government agencies. The challenge of building inclusive communities in STEM is definitely a marathon, rather than a sprint.

**SG:** I think one of the biggest issues is that we’re rarely included in any type of organisational data collection – cultural or organisational experience surveys, for example. Most organisations aren’t even aware of how many LGBTQIA+ researchers are in their own organisation, and what specific issues queer scientists face. As a result, these issues aren’t getting solved. Does your organisation have clear name change procedures? Do you have non-binary options (which aren’t “not-specified” or “other”!) in your onboarding process? Do you have clear policies in place to deal with - and prevent - queerphobic behaviour? And are your senior leaders (the laboratory heads and research managers) trained in these practices? I think if we get some of this institutional behaviour right, then from there we can collectively start to deal with the bigger structural issues at play, such as how to make sure published works can be updated to reflect name changes. We’re making headway in some of these areas, but we need immediate changes to happen at the local, institutional level to ensure LGBTQIA+ people remain in STEM.

*This interview was conducted by Senior Editor George Inglis*.

